# 2-[(2-Carboxy­phen­yl)disulfan­yl]benzoic acid–4,4′-bipyridyl *N*,*N*′-dioxide (1/2)

**DOI:** 10.1107/S1600536810018775

**Published:** 2010-05-26

**Authors:** Rodolfo Moreno-Fuquen, Javier Ellena, Carlos A. De Simone, Leandro Ribeiro, Regina Helena De Almeida Santos

**Affiliations:** a Departamento de Química - Facultad de Ciencias, Universidad del Valle, Apartado 25360, Santiago de Cali, Colombia; b Instituto de Física de São Carlos, IFSC, Universidade de São Paulo, USP, São Carlos, SP, Brazil; c Instituto de Qυ’imica de São Carlos, IFSC, Universidade de São Paulo, USP, São Carlos, SP, Brazil

## Abstract

In the title 2:1 adduct, C_14_H_10_O_4_S_2_·0.5C_10_H_8_N_2_O_2_, which arose from an unexpected oxidation of a precursor, the dihedral angle between the aromatic rings in the disulfide is 82.51 (11)°.  In the crystal, the molecules are linked by O—H⋯O, O—H⋯N and C—H⋯O interactions, generating sheets.

## Related literature

For structural studies of 4,4′-bipyridyl *N*,*N*′-dioxide, see: Lou & Huang (2007 ▶); Reddy *et al.* (2006 ▶). For the disulfide bond in polypeptide chains, see: Gortner & Hoffman (1941 ▶). For a related structure, see: Moreno-Fuquen *et al.* (2003 ▶). For hydrogen bonding, see: Etter (1990 ▶); Nardelli (1995 ▶). 
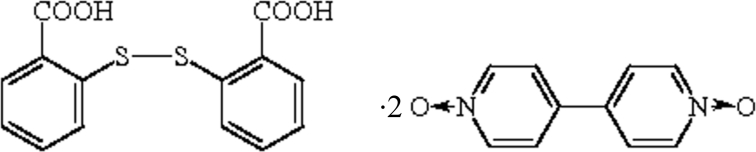


## Experimental

### 

#### Crystal data

C_14_H_10_O_4_S_2_·0.5C_10_H_8_N_2_O_2_
*M* *_r_* = 400.45
Monoclinic, 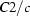

*a* = 21.314 (2) Å
*b* = 10.5621 (8) Å
*c* = 16.005 (8) Å
β = 105.412 (8)°
*V* = 3473.5 (18) Å^3^
*Z* = 8
Mo *K*α radiation
μ = 0.34 mm^−1^
*T* = 291 K
0.22 × 0.18 × 0.12 mm

#### Data collection

Rigaku AFC-7S diffractometer
Absorption correction: ψ scan (North *et al.*, 1968 ▶) *T*_min_ = 0.951, *T*_max_ = 0.990
3066 measured reflections
2781 independent reflections
2658 reflections with *I* > 2σ(*I*)
*R*_int_ = 0.046
3 standard reflections every 120 min  intensity decay: 0.9%

#### Refinement

*R*[*F* ^2^ > 2σ(*F* ^2^)] = 0.065
*wR*(*F* ^2^) = 0.192
*S* = 1.11
2781 reflections
244 parameters
H-atom parameters constrained
Δρ_max_ = 0.74 e Å^−3^
Δρ_min_ = −0.47 e Å^−3^

### 

Data collection: *MSC/AFC Diffractometer Control Software* (Molecular Structure Corporation, 1993 ▶); cell refinement: *MSC/AFC Diffractometer Control Software*; data reduction: *TEXSAN* (Molecular Structure Corporation, 1995 ▶); program(s) used to solve structure: *SHELXS97* (Sheldrick, 2008 ▶); program(s) used to refine structure: *SHELXL97* (Sheldrick, 2008 ▶); molecular graphics: *ORTEP-3 for Windows* (Farrugia, 1997 ▶) and *Mercury* (Macrae *et al.*, 2006 ▶); software used to prepare material for publication: *WinGX* (Farrugia, 1999 ▶).

## Supplementary Material

Crystal structure: contains datablocks I, global. DOI: 10.1107/S1600536810018775/hg2676sup1.cif

Structure factors: contains datablocks I. DOI: 10.1107/S1600536810018775/hg2676Isup2.hkl

Additional supplementary materials:  crystallographic information; 3D view; checkCIF report

## Figures and Tables

**Table 1:** Hydrogen-bond geometry (Å, °)

*D*—H⋯*A*	*D*—H	H⋯*A*	*D*⋯*A*	*D*—H⋯*A*
O5—H55⋯O1^i^	0.82	1.77	2.583 (3)	174
O3—H3⋯O1^ii^	0.82	1.86	2.672 (3)	170
O5—H55⋯N1^i^	0.82	2.50	3.255 (3)	154
C17—H17⋯O2^iii^	0.93	2.59	3.359 (4)	140

Symmetry codes: (i) 
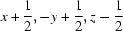
; (ii) 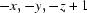; (iii) 
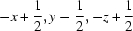
.

**Fig. 1.**
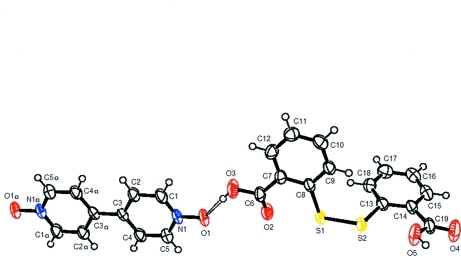
An ORTEP-3 (Farrugia, 1997) plot of the title (I) compound, with the atomic labelling scheme. The shapes of the ellipsoids correspond to 50% probability contours of atomic displacement and, for the sake of clarity, H atoms are shown as spheres of arbitrary radius.

**Fig. 2.**
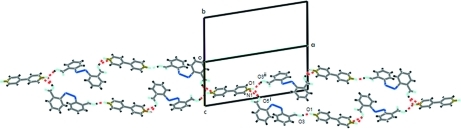
The packing in the unit cell of (I) parallel to the (1 0 -2) plane, showing the R76(57) ring. Hydrogen-bonding interactions are presented as broken lines. Symmetry code: (i) x+1/2,-y+1/2,+z-1/2; (ii) -x,-y,-z+1.

**Fig. 3.**
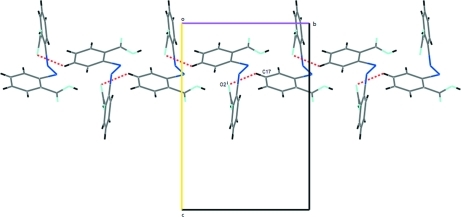
The packing in the unit cell of (I) along [100], showing the formation of C(11) infinite chains. Hydrogen-bonding interactions are presented as broken lines. Symmetry code: (i) -x+1/2,+y-1/2,-z+1/2.

## supplementary crystallographic information

### Comment

The title compound, C_14_H_10_O_4_S_2_, 0.5(C_10_H_8_N_2_O_2_), (I), belongs to a series of molecular systems based on 4,4'-bipyridyl N,*N*'-dioxide (DPNO) with diverse hydrogen-bond donors, that has been synthesized in our research group (Moreno-Fuquen *et al.*, 2003). Several authors have reported the formation of co-crystals from DPNO moiety (Lou & Huang, 2007; Reddy *et al.*, 2006), taking advantage of the strong acceptor character of the N-oxide group. Initially, it was unclear which intermolecular hydrogen bond is formed: S—H ··· O or O—H ··· O. The oxidation of sulfhydryl (S—H) group, of the 2-mercaptobenzoic acid (MBA), allows the formation of 2,2'-dicarboxyphenyldisulfide molecule (CPS), which enters in the reaction with DPNO to form the title co-crystal. The strong S—S disulfide bond formed in this structure, is important in linking polypeptide chains of proteins (Gortner & Hoffman, 1941). A perspective view of the molecule of the title compound, showing the atomic numbering scheme, is given in Fig. 1. The DPNO and CPS molecules are held together by an intermolecular hydrogen bonds between the O1 atom of the N-oxide group of DPNO and the O5 and O3 of the CPS molecule, with O···O distances of 2.583 (3) and 2.672 (3) Å respectively. The central S1—S2 bond length is 2.0397 (10) Å and the Car-S—S-Car torsion angle is -86.15 (14)%. There are no intramolecular O—H ··· S bonds in the structure. It is noted however, that carboxylic groups of the CPS molecule, exhibit different behaviors with respect to the presence of the neighboring sulfur atom. Indeed, while one of the O—H group of carboxylic group is oriented away from the S1 atom [torsion angle C13 C14 C19 O5, -12.7 (5)°], the second O—H group is oriented near to S2 atom [torsion angle C8 C7 C6 O3 163.8 (3)]. the DPNO molecule is almost coplanar with one of the planes of the CPS molecule showing a dihedral angle of 0.71 (7)°. With the other plane of CPS, the DPNO molecule forms a dihedral angle of 82.52 (11)°. The growth of the crystal system can be explained through a hydrogen bonding scheme (Table 1) (Nardelli, 1995). The title molecule is characterized by the formation of O—H···O and O—H···N hydrogen bonds and other weak C—H···O interactions. In a first substructure atom O5 in the molecule at (x+1/2,-y+1/2,+z-1/2) and atom O3 in the molecule at (-x,-y,-z+1) act simultaneously as hydrogen bond donors to O1 atom in the molecule at (x,y,z). In turn, the O5 atom is linked to the N1 atom at (x,y,z). The propagation of these interactions forms a large R^7^_6_(57) ring (Etter, 1990) in the (1 0 -2) plane (Fig. 2). In a second substructure, atom C17 in the molecule at (x,y,z) acts as hydrogen bond donor to O2 atom in the molecule at -x+1/2,+y-1/2,-z+1/2. The propagation of this interaction forms C(11) continuous chains and running along [010] direction. All of these interactions define an infinite two-dimensional network for the structure (I) (Fig. 3).

### Experimental

The sinthesis of the title compound (I) was carried out by slow evaporation of equimolar quantities of 2-mercaptobenzoic acid (0.537 g., 0.0035 mol) and 4,4'-bipyridyl N,*N*'-dioxide (0.655 g) in 50 ml of dry acetonitrile. Pale-yellow prisms of good quality, suitable for X-ray analysis were obtained. The initial reagents were purchased from Aldrich Chemical Co. and were used as received.

### Refinement

All H-atoms were located from difference maps and were positioned geometrically and refined using a riding model with C–H= 0.93–0.97 Å and Uiso(H)= 1.2U_eq_(C).

### Crystal data

**Table:**

C_14_H_10_O_4_S_2_·0.5C_10_H_8_N_2_O_2_	*F*(000) = 1656
*M**_r_* = 400.45	*D*_x_ = 1.532 Mg m^−^^3^
Monoclinic, *C*2/*c*	Mo *K*α radiation, λ = 0.71073 Å
Hall symbol: -c 2yc	Cell parameters from 25 reflections
*a* = 21.314 (2) Å	θ = 10.3–19.1°
*b* = 10.5621 (8) Å	µ = 0.34 mm^−^^1^
*c* = 16.005 (8) Å	*T* = 291 K
β = 105.412 (8)°	Prism, pale-yellow
*V* = 3473.5 (18) Å^3^	0.22 × 0.18 × 0.12 mm
*Z* = 8	

### Data collection

**Table:**

Rigaku AFC-7S diffractometer	2658 reflections with *I* > 2σ(*I*)
Radiation source: fine-focus sealed X-ray tube	*R*_int_ = 0.046
graphite	θ_max_ = 25.1°, θ_min_ = 2.0°
ω/2θ scans	*h* = −25→24
Absorption correction: ψ scan (North *et al.*, 1968)	*k* = 0→12
*T*_min_ = 0.951, *T*_max_ = 0.990	*l* = 0→19
3066 measured reflections	3 standard reflections every 120 min
2781 independent reflections	intensity decay: 0.9%

### Refinement

**Table:**

Refinement on *F*^2^	Secondary atom site location: difference Fourier map
Least-squares matrix: full	Hydrogen site location: inferred from neighbouring sites
*R*[*F*^2^ > 2σ(*F*^2^)] = 0.065	H-atom parameters constrained
*wR*(*F*^2^) = 0.192	*w* = 1/[σ^2^(*F*_o_^2^) + (0.1415*P*)^2^ + 4.1121*P*] where *P* = (*F*_o_^2^ + 2*F*_c_^2^)/3
*S* = 1.11	(Δ/σ)_max_ < 0.001
2781 reflections	Δρ_max_ = 0.74 e Å^−^^3^
244 parameters	Δρ_min_ = −0.47 e Å^−^^3^
0 restraints	Extinction correction: *SHELXL97* (Sheldrick, 2008)
Primary atom site location: structure-invariant direct methods	Extinction coefficient: none

### Special details

**Table:**

Geometry. All esds (except the esd in the dihedral angle between two l.s. planes) are estimated using the full covariance matrix. The cell esds are taken into account individually in the estimation of esds in distances, angles and torsion angles; correlations between esds in cell parameters are only used when they are defined by crystal symmetry. An approximate (isotropic) treatment of cell esds is used for estimating esds involving l.s. planes.
Refinement. Refinement of *F*^2^ against ALL reflections. The weighted *R*-factor wR and goodness of fit *S* are based on *F*^2^, conventional *R*-factors *R* are based on *F*, with *F* set to zero for negative *F*^2^. The threshold expression of *F*^2^ > σ(*F*^2^) is used only for calculating *R*-factors(gt) etc. and is not relevant to the choice of reflections for refinement. *R*-factors based on *F*^2^ are statistically about twice as large as those based on *F*, and *R*- factors based on ALL data will be even larger.

### Fractional atomic coordinates and isotropic or equivalent isotropic displacement parameters (Å^2^)

**Table:**

	*x*	*y*	*z*	*U*_iso_*/*U*_eq_	
S2	0.34651 (3)	0.02160 (7)	0.27473 (5)	0.0381 (3)	
S1	0.25516 (3)	−0.05076 (8)	0.22805 (5)	0.0389 (3)	
O2	0.13221 (10)	−0.1328 (3)	0.17027 (17)	0.0487 (6)	
O4	0.55073 (13)	0.0281 (3)	0.4215 (2)	0.0761 (9)	
O3	0.07037 (11)	−0.1051 (3)	0.03538 (17)	0.0589 (7)	
H3	0.0408	−0.1159	0.0585	0.088*	
O5	0.46308 (12)	0.1178 (2)	0.3372 (2)	0.0690 (9)	
H55	0.4829	0.1832	0.3551	0.103*	
C13	0.39868 (14)	−0.1151 (3)	0.2968 (2)	0.0359 (7)	
C17	0.41418 (17)	−0.3405 (3)	0.2889 (2)	0.0472 (8)	
H17	0.3975	−0.4197	0.2693	0.057*	
C9	0.29901 (14)	−0.0579 (3)	0.0790 (2)	0.0374 (7)	
H9	0.3401	−0.0404	0.1150	0.045*	
C7	0.18433 (13)	−0.0960 (2)	0.0587 (2)	0.0337 (7)	
C14	0.46425 (14)	−0.1032 (3)	0.3434 (2)	0.0379 (7)	
C11	0.23059 (17)	−0.0955 (3)	−0.0634 (2)	0.0443 (8)	
H11	0.2254	−0.1039	−0.1227	0.053*	
C8	0.24617 (13)	−0.0705 (3)	0.1146 (2)	0.0335 (7)	
C10	0.29099 (16)	−0.0710 (3)	−0.0088 (2)	0.0428 (8)	
H10	0.3268	−0.0632	−0.0314	0.051*	
C18	0.37493 (15)	−0.2348 (3)	0.2689 (2)	0.0421 (7)	
H18	0.3320	−0.2438	0.2363	0.051*	
C19	0.49747 (14)	0.0205 (3)	0.3733 (2)	0.0441 (8)	
C6	0.12735 (14)	−0.1140 (3)	0.0947 (2)	0.0395 (8)	
C15	0.50222 (15)	−0.2117 (3)	0.3643 (2)	0.0455 (8)	
H15	0.5452	−0.2041	0.3969	0.055*	
C16	0.47782 (16)	−0.3297 (3)	0.3378 (2)	0.0480 (8)	
H16	0.5038	−0.4012	0.3527	0.058*	
C12	0.17762 (15)	−0.1077 (3)	−0.0295 (2)	0.0422 (8)	
H12	0.1368	−0.1239	−0.0665	0.051*	
N1	0.08340 (11)	0.1895 (2)	0.91617 (17)	0.0361 (6)	
O1	0.01985 (9)	0.1677 (2)	0.88364 (16)	0.0460 (6)	
C4	0.18672 (14)	0.2206 (3)	0.8937 (2)	0.0399 (7)	
H4	0.2127	0.2256	0.8555	0.048*	
C1	0.10782 (14)	0.2062 (3)	1.0006 (2)	0.0449 (8)	
H1	0.0804	0.2023	1.0371	0.054*	
C3	0.21476 (12)	0.2373 (2)	0.98169 (19)	0.0311 (6)	
C2	0.17312 (14)	0.2295 (3)	1.0351 (2)	0.0436 (8)	
H2	0.1895	0.2400	1.0946	0.052*	
C5	0.12157 (14)	0.1970 (3)	0.8619 (2)	0.0428 (7)	
H5	0.1038	0.1863	0.8027	0.051*	

### Atomic displacement parameters (Å^2^)

**Table:**

	*U*^11^	*U*^22^	*U*^33^	*U*^12^	*U*^13^	*U*^23^
S2	0.0244 (4)	0.0417 (5)	0.0448 (6)	−0.0003 (3)	0.0033 (3)	−0.0071 (3)
S1	0.0220 (4)	0.0573 (5)	0.0375 (6)	−0.0022 (3)	0.0081 (3)	−0.0064 (3)
O2	0.0295 (11)	0.0734 (15)	0.0435 (17)	−0.0045 (10)	0.0101 (10)	0.0060 (11)
O4	0.0343 (15)	0.0647 (17)	0.109 (3)	−0.0041 (11)	−0.0167 (15)	−0.0058 (16)
O3	0.0253 (12)	0.092 (2)	0.0547 (16)	−0.0101 (11)	0.0029 (10)	0.0050 (13)
O5	0.0371 (13)	0.0452 (13)	0.105 (2)	−0.0065 (10)	−0.0149 (13)	−0.0014 (13)
C13	0.0301 (14)	0.0420 (15)	0.0366 (18)	0.0015 (11)	0.0104 (12)	0.0001 (12)
C17	0.056 (2)	0.0402 (16)	0.050 (2)	−0.0028 (14)	0.0208 (16)	−0.0012 (13)
C9	0.0296 (15)	0.0405 (15)	0.044 (2)	−0.0018 (11)	0.0129 (13)	−0.0009 (12)
C7	0.0281 (14)	0.0313 (13)	0.040 (2)	−0.0027 (10)	0.0060 (12)	0.0008 (11)
C14	0.0269 (14)	0.0446 (16)	0.044 (2)	0.0017 (11)	0.0127 (12)	0.0010 (13)
C11	0.054 (2)	0.0433 (16)	0.038 (2)	−0.0037 (14)	0.0158 (15)	−0.0013 (13)
C8	0.0247 (13)	0.0328 (13)	0.044 (2)	0.0005 (10)	0.0103 (12)	−0.0008 (11)
C10	0.0435 (17)	0.0418 (16)	0.050 (2)	−0.0021 (13)	0.0243 (15)	0.0017 (13)
C18	0.0367 (15)	0.0448 (16)	0.045 (2)	−0.0064 (12)	0.0107 (13)	−0.0047 (13)
C19	0.0232 (16)	0.0506 (18)	0.057 (2)	−0.0011 (12)	0.0079 (14)	−0.0019 (14)
C6	0.0256 (14)	0.0360 (15)	0.054 (2)	−0.0054 (11)	0.0055 (13)	−0.0040 (13)
C15	0.0336 (15)	0.0553 (19)	0.050 (2)	0.0077 (13)	0.0142 (13)	0.0056 (15)
C16	0.0466 (18)	0.0494 (18)	0.053 (2)	0.0115 (14)	0.0214 (15)	0.0080 (14)
C12	0.0401 (17)	0.0398 (16)	0.043 (2)	−0.0081 (12)	0.0049 (14)	−0.0055 (12)
N1	0.0223 (11)	0.0340 (12)	0.0523 (19)	0.0035 (9)	0.0108 (11)	0.0060 (10)
O1	0.0191 (10)	0.0503 (13)	0.0661 (16)	0.0003 (8)	0.0071 (9)	0.0062 (10)
C4	0.0290 (15)	0.0531 (17)	0.042 (2)	0.0012 (12)	0.0171 (13)	0.0015 (13)
C1	0.0281 (14)	0.065 (2)	0.047 (2)	−0.0006 (13)	0.0187 (14)	0.0026 (15)
C3	0.0253 (14)	0.0300 (12)	0.0416 (19)	0.0032 (10)	0.0152 (12)	0.0025 (11)
C2	0.0277 (14)	0.065 (2)	0.042 (2)	−0.0003 (13)	0.0157 (13)	−0.0003 (14)
C5	0.0316 (15)	0.0500 (17)	0.047 (2)	0.0025 (13)	0.0108 (14)	0.0025 (14)

### Geometric parameters (Å, °)

**Table:**

S2—C13	1.799 (3)	C11—C10	1.375 (5)
S2—S1	2.0397 (10)	C11—C12	1.383 (5)
S1—C8	1.786 (3)	C11—H11	0.9300
O2—C6	1.203 (4)	C10—H10	0.9300
O4—C19	1.194 (4)	C18—H18	0.9300
O3—C6	1.331 (4)	C15—C16	1.373 (5)
O3—H3	0.8200	C15—H15	0.9300
O5—C19	1.305 (4)	C16—H16	0.9300
O5—H55	0.8200	C12—H12	0.9300
C13—C18	1.391 (4)	N1—C1	1.324 (4)
C13—C14	1.404 (4)	N1—O1	1.336 (3)
C17—C16	1.379 (5)	N1—C5	1.340 (4)
C17—C18	1.380 (5)	C4—C5	1.369 (4)
C17—H17	0.9300	C4—C3	1.387 (5)
C9—C10	1.376 (5)	C4—H4	0.9300
C9—C8	1.397 (4)	C1—C2	1.376 (4)
C9—H9	0.9300	C1—H1	0.9300
C7—C12	1.386 (5)	C3—C2	1.389 (4)
C7—C8	1.408 (4)	C3—C3^i^	1.484 (5)
C7—C6	1.488 (4)	C2—H2	0.9300
C14—C15	1.391 (4)	C5—H5	0.9300
C14—C19	1.502 (4)		
			
C13—S2—S1	104.55 (10)	O4—C19—C14	123.5 (3)
C8—S1—S2	104.49 (9)	O5—C19—C14	112.5 (3)
C6—O3—H3	109.5	O2—C6—O3	123.2 (3)
C19—O5—H55	109.5	O2—C6—C7	123.3 (3)
C18—C13—C14	118.6 (3)	O3—C6—C7	113.5 (3)
C18—C13—S2	120.9 (2)	C16—C15—C14	121.7 (3)
C14—C13—S2	120.6 (2)	C16—C15—H15	119.2
C16—C17—C18	120.6 (3)	C14—C15—H15	119.2
C16—C17—H17	119.7	C15—C16—C17	119.0 (3)
C18—C17—H17	119.7	C15—C16—H16	120.5
C10—C9—C8	120.8 (3)	C17—C16—H16	120.5
C10—C9—H9	119.6	C11—C12—C7	121.2 (3)
C8—C9—H9	119.6	C11—C12—H12	119.4
C12—C7—C8	119.3 (3)	C7—C12—H12	119.4
C12—C7—C6	120.6 (3)	C1—N1—O1	120.2 (2)
C8—C7—C6	120.1 (3)	C1—N1—C5	120.7 (3)
C15—C14—C13	119.2 (3)	O1—N1—C5	119.0 (3)
C15—C14—C19	116.4 (3)	C5—C4—C3	121.4 (3)
C13—C14—C19	124.4 (3)	C5—C4—H4	119.3
C10—C11—C12	119.5 (3)	C3—C4—H4	119.3
C10—C11—H11	120.3	N1—C1—C2	121.0 (3)
C12—C11—H11	120.3	N1—C1—H1	119.5
C9—C8—C7	118.6 (3)	C2—C1—H1	119.5
C9—C8—S1	121.5 (2)	C4—C3—C2	116.3 (3)
C7—C8—S1	119.8 (2)	C4—C3—C3^i^	122.8 (3)
C11—C10—C9	120.6 (3)	C2—C3—C3^i^	120.9 (3)
C11—C10—H10	119.7	C1—C2—C3	120.5 (3)
C9—C10—H10	119.7	C1—C2—H2	119.8
C17—C18—C13	120.9 (3)	C3—C2—H2	119.8
C17—C18—H18	119.5	N1—C5—C4	120.0 (3)
C13—C18—H18	119.5	N1—C5—H5	120.0
O4—C19—O5	123.9 (3)	C4—C5—H5	120.0
			
C13—S2—S1—C8	−86.15 (14)	C13—C14—C19—O5	−12.7 (5)
S1—S2—C13—C18	10.1 (3)	C12—C7—C6—O2	163.0 (3)
S1—S2—C13—C14	−169.3 (2)	C8—C7—C6—O2	−15.1 (4)
C18—C13—C14—C15	−3.6 (5)	C12—C7—C6—O3	−18.1 (4)
S2—C13—C14—C15	175.9 (2)	C8—C7—C6—O3	163.8 (3)
C18—C13—C14—C19	176.5 (3)	C13—C14—C15—C16	2.3 (5)
S2—C13—C14—C19	−4.1 (4)	C19—C14—C15—C16	−177.7 (3)
C10—C9—C8—C7	−0.5 (4)	C14—C15—C16—C17	0.4 (5)
C10—C9—C8—S1	−178.8 (2)	C18—C17—C16—C15	−1.9 (5)
C12—C7—C8—C9	−0.1 (4)	C10—C11—C12—C7	−0.2 (5)
C6—C7—C8—C9	178.1 (3)	C8—C7—C12—C11	0.4 (4)
C12—C7—C8—S1	178.2 (2)	C6—C7—C12—C11	−177.7 (3)
C6—C7—C8—S1	−3.6 (4)	O1—N1—C1—C2	−179.6 (3)
S2—S1—C8—C9	10.4 (2)	C5—N1—C1—C2	−1.3 (5)
S2—S1—C8—C7	−167.9 (2)	C5—C4—C3—C2	−0.4 (4)
C12—C11—C10—C9	−0.3 (5)	C5—C4—C3—C3^i^	179.8 (3)
C8—C9—C10—C11	0.7 (5)	N1—C1—C2—C3	0.8 (5)
C16—C17—C18—C13	0.5 (5)	C4—C3—C2—C1	0.1 (5)
C14—C13—C18—C17	2.2 (5)	C3^i^—C3—C2—C1	179.8 (3)
S2—C13—C18—C17	−177.2 (3)	C1—N1—C5—C4	0.9 (5)
C15—C14—C19—O4	−9.3 (5)	O1—N1—C5—C4	179.3 (3)
C13—C14—C19—O4	170.6 (4)	C3—C4—C5—N1	−0.1 (5)
C15—C14—C19—O5	167.4 (3)		

Symmetry codes: (i) −*x*+1/2, −*y*+1/2, −*z*+2.

### Hydrogen-bond geometry (Å, °)

**Table:**

*D*—H···*A*	*D*—H	H···*A*	*D*···*A*	*D*—H···*A*
O5—H55···O1^ii^	0.82	1.77	2.583 (3)	174
O3—H3···O1^iii^	0.82	1.86	2.672 (3)	170
O5—H55···N1^ii^	0.82	2.50	3.255 (3)	154
C17—H17···O2^iv^	0.93	2.59	3.359 (4)	140

Symmetry codes: (ii) *x*+1/2, −*y*+1/2, *z*−1/2; (iii) −*x*, −*y*, −*z*+1; (iv) −*x*+1/2, *y*−1/2, −*z*+1/2.
